# Self-Compassion and Its Association With Ruminative Tendencies and Vagally Mediated Heart Rate Variability in Recurrent Major Depression

**DOI:** 10.3389/fpsyg.2022.798914

**Published:** 2022-03-07

**Authors:** Julie Lillebostad Svendsen, Elisabeth Schanche, Jon Vøllestad, Endre Visted, Sebastian Jentschke, Anke Karl, Per-Einar Binder, Berge Osnes, Lin Sørensen

**Affiliations:** ^1^Bjørgvin District Psychiatric Centre, Haukeland University Hospital, Bergen, Norway; ^2^Department of Clinical Psychology, University of Bergen, Bergen, Norway; ^3^Department of Psychosocial Science, University of Bergen, Bergen, Norway; ^4^Mood Disorder Centre, College of Life and Environmental Sciences, University of Exeter, Exeter, United Kingdom; ^5^Department of Biological and Medical Psychology, University of Bergen, Bergen, Norway

**Keywords:** self-compassion, recurrent depression, rumination, vagally mediated heart rate variability, vagal (parasympathetic) reactivity

## Abstract

**Background:**

Recurrent Major Depressive Disorder (MDD) is one of the most disabling mental disorders in modern society. Prior research has shown that self-compassion protects against ruminative tendencies, a key feature of recurrent MDD. In addition, self-compassion has been found to be positively related to higher psychophysiological flexibility (indexed by a higher vagally mediated heart rate variability; vmHRV) in young, healthy adults. To our knowledge, there is a lack of studies on how self-compassion relates to vmHRV in patients with recurrent MDD. The aim of the current study was to investigate whether higher self-compassion would associate with (1) lower ruminative tendencies and (2) higher vmHRV in a sample of adults with recurrent MDD.

**Methods:**

We included a sample of 63 patients (46 females) between 20 and 71 years old (*M* = 40.24, *SD* = 12.8) with a history of three or more depressive episodes. They filled out the Self-Compassion Scale (SCS), Beck Depression Inventory (BDI), and Rumination Rating Scale (RRS). ECG (used to derive vmHRV) was acquired while resting and the square root of the mean squared differences of successive RR interval values (RMSSD) was calculated as measure of vmHRV.

**Results:**

As hypothesized, self-compassion was associated with lower ruminative tendencies. However, self-compassion was not associated with level of vmHRV. Several confounding variables were controlled for in the statistical analyses, and higher age predicted lower vmHRV across all statistical analyses.

**Conclusion:**

The results confirmed our hypothesis that higher self-compassion would be associated with lower ruminative tendencies in recurrent MDD. Contrary to our expectation, we did not find that the tendency to be more self-compassionate was associated with higher vmHRV. As such, higher self-compassion seems to relate with a lower tendency to ruminate about past mistakes and events but does not seem to relate to a flexible autonomic stress response (as indexed by higher vmHRV). Other potential explanatory factors for lower vmHRV in recurrent MDD is suggested as focus for exploration in future studies.

## Introduction

Recurrent Major Depressive Disorder (MDD) is one of the most disabling and costly mental disorders in modern society (Layard, 2006; Collins et al., 2011). Adults with recurrent MDD have experienced several major depressive episodes and have a high risk of new episodes (e.g., Solomon et al., 2000). A central vulnerability factor for relapse of major depressive episodes and thus for a chronic course of major depression is the tendency to ruminate about past mistakes and negative emotions (Nolen-Hoeksema et al., 2008). Such negative rumination typically sets the stage for vicious circles, in which automatic self-critical thinking activates sad mood, which in turn reinforces and fuels the ruminative self-attacks and emotional reactivity (stress) responses in the body (Segal et al., 1996). Several studies have shown that ruminative tendencies are associated with an index of the autonomic nervous system’s ability to handle emotional stress—vagally mediated heart rate variability (vmHRV; Thayer and Lane, 2000, 2009; Appelhans and Luecken, 2006), both in healthy adult samples (e.g., Ottaviani et al., 2016; Williams et al., 2017; Carnevali et al., 2018; Dell’Acqua et al., 2021) as well as in adults suffering from major depression (Woody et al., 2014). However, there is a lack of studies on the role of protective psychological factors such as self-compassion on the level of vmHRV in the depression literature. This is of interest since self-compassionate individuals with MDD typically have less ruminative tendencies (Krieger et al., 2013).

A meta-analysis showed that adults with major depression typically have lower levels of vmHRV than healthy controls (Kemp et al., 2010). Within the clinical group, increasing symptom severity of depression was associated with decreasing vmHRV. This meta-analysis further controlled for the effect of cardiovascular diseases on vmHRV since a chronic course of depression is a known risk factor for cardiovascular diseases (Katon et al., 2004; Dhar and Barton, 2016). However, the use of antidepressant medication, including long-term use, appeared not to affect the named relation between increasing symptom severity and decreasing vmHRV. It is of interest to explore psychological processes that may serve to counteract the maladaptive cognitive, emotional, and psychophysiological dynamics seen in recurrent MDD. Self-compassion is suggested to protect against the negative effect of self-critical rumination and depressive mood on the autonomic nervous system (Gilbert, 2009).

Being self-compassionate involves extending kindness and support toward oneself when faced with difficult situations or perceived failure (Neff, 2003b). Rather than judging and attacking oneself in times of failure, one actively soothes and reassures oneself, and tries to view difficulties as a universal part of life instead of proof of personal weakness or flaws (Neff, 2003b). Treating oneself with compassion when in low mood is suggested to have similar positive effects on the autonomic nervous system as receiving comfort from another person, for example an attachment figure (Gilbert, 2005). This self-soothing attitude and action may disrupt the chronic stress activation associated with recurrent MDD through activating feelings of safety and comfort (Gilbert, 2009; Kuyken et al., 2010). Thus, inner self-talk characterized by reassuring and supportive messages and a warm tone may calm emotional stress responses, and make the person feel safe and comforted. The soothing effect is thought to increase vagal stimulation on the parasympathetic branch of the autonomic nervous system, leading to higher vmHRV. The parasympathetic branch has a shorter response latency than the sympathetic branch, thereby producing rapid beat-to-beat changes (see Appelhans and Luecken, 2006; Thayer and Lane, 2009). Lower vmHRV marks a lower vagal contribution to beat-to-beat changes and is shown to be associated with increased risk of cardiovascular events and all-cause death (Hillebrand et al., 2013; Kubota et al., 2017; Fang et al., 2020). Self-compassion may help people to hold difficult emotions in mindful awareness with acceptance, instead of avoiding or overidentifying with difficult emotions. Moreover, self-compassion may make it easier for the person to frame difficulties in the light of common humanity, normalizing failure instead of taking it personally. As such, self-soothing tendencies in times of difficulty can transform negative emotions into a more positive affective state (Neff, 2003b) and reduce subsequent autonomic stress (see Gilbert (2005), Porges (2007)).

Self-compassion is proposed to act as an antidote to rumination (Neff, 2003b). Although ruminating typically feels productive to the individual who performs it, research to the contrary indicates that it prevents effective emotion regulation. This is because it involves an experiential avoidance, where one avoids disturbing emotions such as sadness through suppressing or disconnecting from it (Thomas et al., 2015). Self-compassion on the other hand, is regarded as an effective emotion regulation strategy (e.g., Neff, 2003b; Diedrich et al., 2014; Finlay-Jones, 2017), allowing uncomfortable feelings and sensations to enter awareness. Being self-compassionate is thus suggested to enable less use of rumination as a coping strategy (Neff, 2003b). Indeed, a substantial amount of research has demonstrated that higher self-compassion is related to lower ruminative tendencies. This has been demonstrated both in young healthy adults (Neff, 2003b; Neff et al., 2007; Neff and Vonk, 2009; Raes, 2010), and in clinical, depressive samples (Krieger et al., 2013; Karl et al., 2018; Bakker et al., 2019).

In recent years, several studies have demonstrated that higher levels of self-compassion are also related to a higher flexibility in psychophysiological responding, as measured by higher levels of vmHRV. We have previously shown that in young, healthy adults, higher levels of self-compassion were associated with higher levels of vmHRV (Svendsen et al., 2016). Correspondingly, Luo et al. (2018) found that students with higher levels of dispositional self-compassion had higher levels of vmHRV when performing a stress test (giving a speech) as compared to individuals with lower levels of self-compassion. It has also been shown that training to enhance self-compassion leads to increased levels of vmHRV (Rockliff et al., 2008; Kok et al., 2013; Arch et al., 2014; Matos et al., 2017; Petrocchi et al., 2017; Kirschner et al., 2019; Luo et al., 2020). For example, Kirschner et al. (2019) used an experimental design and found that participants who listened to a sound file aimed at increasing self-compassion had higher vmHRV than control participants. In another interesting recent study, Luo et al. (2020) found that a short compassionate self-talk intervention led to increased vmHRV during a pain exercise (holding a bottle of iced water), as compared to the control condition which did not practice compassionate self-talk. These studies support the protective effects of self-compassion on activating the parasympathetic system. Self-compassion has also been shown to be associated with other biological processes underlying emotional and physiological stress responding, such as level of cortisol (Herriot et al., 2018), level of stress-induced inflammation (Breines et al., 2014), immunological markers (Friis et al., 2015) and stress-induced salivary alpha amylase concentration (Breines et al., 2015). Moreover, a recent study (Proskurnina et al., 2021) found that soothing, pleasant physical touch led to significant increase in salivary oxytocin level (as compared to a control group who did not receive soothing touch), as well as increased vmHRV levels. This indicates that soothing touch is stress-reducing, has anti-inflammatory effects, and facilitates parasympathetic activation.

Despite the findings of self-compassion positively associating with higher vmHRV in healthy populations, no prior study has to our knowledge examined how self-compassion associates with vmHRV in patients with recurrent MDD. This is important to investigate, as previous research has demonstrated self-compassion to be a key protective factor against depressive symptoms (Kuyken et al., 2010; MacBeth and Gumley, 2012). Kuyken et al. (2010) found that increased self-compassion after Mindfulness-based cognitive therapy (MBCT) led to a decoupling of the relationship between reactivity of negative rumination and poor outcome (depressive relapse and severity of depressive symptoms 15 months follow-up). Moreover, the level of self-compassion predicted later levels of depressive symptoms but not vice versa, indicating that lower tendencies of self-compassion are a *cause* and not merely a consequence of depressive symptoms (Krieger et al., 2016).

The aim of the current study was to investigate in a sample of adults with recurrent MDD in full or partial remission whether higher self-compassion was associated with (1) lower levels of ruminative tendencies, and (2) higher levels of vmHRV. Most prior studies investigating the association between self-compassion and ruminative tendencies in clinical samples have included a mixed sample of current, lifetime history of and recurrent MDD (see for instance Krieger et al., 2013; Bakker et al., 2019). Moreover, no prior study has investigated the relation between levels of self-compassion and vmHRV in a sample of recurrent MDD. We included adults with recurrent MDD in full or partial remission to study the effect of a chronic course of depression and not the acute effect of a current depressive episode. Due to recurrent MDD being characterized by heterogeneity, we explored if these associations were influenced by differences within the sample in symptom expression such as number and severity of depressive episodes, age of onset, use of antidepressant and blood pressure medication, and severity of comorbid symptom constellations.

## Materials and Methods

### Participants

The current study used baseline data collected as part of a larger randomized controlled trial of change mechanisms in MBCT for adults with recurrent MDD (see also Schanche et al. (2020, 2021); and the entry of the study in the ISRCT registry: Trial no. ISRCTN18001392).

Adults with recurrent MDD were recruited in three separate cohorts from May 2016 to August 2017. Most participants were recruited through advertisements posted at waiting rooms and offices of general practitioners in the municipality of Bergen, Norway. Information about the project was also posted on forums related to mental health on social media such as Facebook. The advertisements directed participants to a webpage^1^ containing more detailed information about the project, contact information and lists of inclusion and exclusion criteria.

Sixty-eight adults with recurrent MDD were eligible for participation in the current study. Four of these adults withdrew prior to the collection of baseline measures of mental health symptoms and cognitive and psychophysiological functioning (see Schanche et al. (2020)). The final sample with baseline data was *N* = 63. Inclusion criteria were an age of 18 years or older, at least three previous episodes of major depression, and remission from such an episode at the point of inclusion (partial or full). Exclusion criterium was comorbidity with a severe mental disorder (such as a history of schizophrenia, psychosis, or bipolar disorder, or another mental disorder in need of treatment, e.g., borderline personality disorder, posttraumatic stress disorder, severe obsessive-compulsive disorder, severe eating disorder, or current substance use disorder; see Schanche et al. (2020) for full description of inclusion criteria).

Participants gave written informed consent before being enrolled in the study. The study followed the guidelines of the declaration of Helsinki, and also adhered to the CONSORT guidelines. It was approved by the Regional Committee for Medical Research Ethics of South-Eastern Norway (Reference number: 2016/388). Participants received no economic compensation for participation but received the MBCT program free of charge.

### Procedure

#### Diagnostic Assessment

The structured clinical interview M.I.N.I. International Neuropsychiatric Interview (M.I.N.I.; Sheehan et al., 1998) was used to assess diagnoses according to the Diagnostic and Statistical Manual of Mental Disorders-IV (DSM-IV) Axis I. The interview comprises 17 modules including mood disorders, anxiety disorders, psychotic disorders, alcohol, and substance use disorders, eating disorders, and antisocial personality disorder. The borderline subscale of the Structured Clinical Interview (SCID-II; Gibbon et al., 1997) for the DSM-IV was used to screen for borderline personality disorder. Demographic variables, age of onset of depression (assessed by retrospective recall), number of depressive episodes, and time in remission were also assessed during the clinical interviews. Since a majority of the participants had problems remembering exactly how many prior MDD episodes they had experienced, a dichotomous variable of > 10 episodes was applied in the current study. The Hamilton Depression rating scale (Hamilton, 1960) was used to assess level of depression. This is a clinician-administered depression assessment scale containing 17 items capturing symptoms of depression experienced over the past week. The clinical interviews were performed by two clinical psychologists who were Ph.D. students (JLS and EV) and had comprehensive experience and training in diagnostics.

#### Procedure

The collection of the baseline data took approximately 3 h for each of the participants. First, the participants were informed about the study and thereafter gave informed consent to participate. Then, they filled out questionnaires on self-compassion, rumination, depressive symptoms, mindfulness, emotion regulation, childhood trauma, attachment, and personality traits (see Schanche et al. (2020)). Afterward, they performed cognitive tests and heart rate was measured with electrocardiogram (ECG) during rest and a mindfulness instruction of body scan.

### Measures

#### Self-Compassion Scale

The Self-Compassion Scale (SCS; Neff, 2003a) was used to measure the ability to meet oneself with kindness and support when suffering. The SCS consists of 26 items measuring three positive and three negative subscales. The positive subscales are self-kindness, common humanity and mindfulness, and the negative subscales are self-judgment, isolation, and overidentification. Items are rated on a five-point Likert-type scale in which 1 indicates “almost never” and 5 indicates “almost always.” High scores on the positive subscales and low scores on the negative subscales equal an overall high level of self-compassion. The SCS has shown good test-retest reliability (Neff, 2003a) and cross-cultural validity and reliability (Neff et al., 2008). We used a Norwegian validated translation of the SCS (Dundas et al., 2016). In the current study, the level of self-compassion varied from 1.16 to 3.92 with a mean level of 2.57 (*SD* = 0.61). Cronbach’s alpha for SCS total was 0.92. Inter-item correlation ranges were acceptable (see Supplementary Table 1).

#### Forms of Self-Criticizing and Reassuring Scale

The Forms of Self-Criticizing and Reassuring Scale (FSCRS; Gilbert et al., 2004) is a 22-item questionnaire measuring participants thoughts and feelings about themselves when things go wrong for them. The scale comprises three subscales. The “Reassured self” captures the ability to extend feelings of warmth and safeness toward oneself when facing difficulties. The “Inadequate self” captures a sense of feeling internally put down when facing setbacks. The “Hated self” captures a more destructive, disgust-based response to difficulties characterized by self-dislike and an aggressive desire to hurt oneself during failure. The FSCRS has been shown to have good internal consistency and construct validity (Gilbert et al., 2004; Kupeli et al., 2013), sufficient test-retest reliability (Castilho et al., 2015) and is also shown to be congruent with other measures of self-criticism (Gilbert et al., 2004). In the current study we used a Norwegian translation (Stiegler et al., 2018). In the current study, scores on “Reassured self” ranged from 8 to 35, with a mean level of 19.61 (*SD* = 5.46), “Inadequate self” ranged from 9 to 45, with a mean level of 32.06 (*SD* = 7.13) and scores on “Hated self” ranged from 5 to 25 with a mean level of 11.67 (*SD* = 4.59). Cronbach’s alphas for FSCRS “Reassured self,” “Inadequate self,” and “Hated self” were 0.85, 0.83, and 0.83, respectively.

#### Rumination-Reflection Questionnaire

The 12-item rumination subscale of the Rumination-Reflection Questionnaire (RRQ-Rum; Trapnell and Campbell, 1999) was used to measure ruminative tendencies. The responses are given on a five-point Likert type scale where 1 indicates “strongly disagree” and 5 indicates “strongly agree.” The RRQ-Rum scale has been shown to have a high internal reliability (Cronbach’s α = 0.91; cf. Verplanken et al., 2007), and adequate test-retest reliability (Trapnell and Campbell, 1999). We used a Norwegian translation of the RRQ-Rum (Verplanken et al., 2007). In the current study, the level of ruminative tendencies ranged from 1.67 to 4.75 with a mean level of 3.81 (*SD* = 0.59). Cronbach’s alpha for RRQ-rum was 0.92, and inter-item correlation ranges were acceptable (see Supplementary Table 2).

#### Ruminative Response Scale-Short Form

The 10-item brooding subscale of the Ruminative Response Scale-Short Form (RRS-short form; Treynor et al., 2003) was used to assess ruminative tendencies. This scale aims to measure styles of responding when experiencing negative emotions. Responses are given on a four-point Likert scale in which 1 indicates “almost never” and 4 indicates “almost always.” The scale consists of two subscales. RRS reflection measures engagement with voluntary cognitive-analytical problem-solving in order to alleviate depressive symptoms. The RRS brooding measures wallowing and sulking; a passive comparison of one’s current situation with an unachieved standard. The scale has shown good reliability and validity in depressed samples (Parola et al., 2017), and has shown good test-retest reliability (Treynor et al., 2003). In the current study, the level of brooding rumination ranged from 8.00 to 20.00 with a mean of 13.48 (*SD* = 3.09). Cronbach’s alpha for RRS brooding was 0.68.

#### Beck Depression Inventory II

The Beck Depression Inventory II (BDI-II; Beck et al., 1996) was used to measure level of depressive symptoms during the past 2 weeks. BDI-II comprises 21 items assessing core symptoms of depression, such as loss of energy, sad mood, and feelings of hopelessness. Answers are rated on a four-point Likert scale ranging from 0 to 3, and higher scores on BDI-II indicate more severe depressive symptoms. The scale is shown to have acceptable convergent and discriminant validity as well as good test-retest reliability (Beck et al., 1996; Wang and Gorenstein, 2013). In the current study we used a Norwegian translation (Aasen, 2001). In the current study, the level of BDI-II ranged from 0.00 to 30.00 with a mean of 12.63 (*SD* = 8.03). In total, 13 individuals had BDI total scores above cut-off score moderately depressed (as defined by BDI total scores between 20 and 28; Aasen, 2001) and two individuals had BDI total scores above cut-off score for severely depressed (as defined by a BDI score over 28; Aasen, 2001). Cronbach’s alpha for BDI was 0.88.

#### Heart Rate Variability

Heart rate variability data were obtained from electrocardiography (ECG) signals. The ECG signals were recorded using a standard 12-lead resting ECG where participants were lying in a relaxed, supine position in a quiet environment, following established standard guidelines (Task Force of the European Society of Cardiology and the North American Society of Pacing and Electrophysiology, 1996). We chose to use resting levels of vmHRV because this is the condition that have been highlighted to be a trait-marker of flexibility in psychophysiological regulation in theories of vmHRV (Appelhans and Luecken, 2006; Thayer and Lane, 2009; Thayer et al., 2009; Beauchaine and Thayer, 2015; Ottaviani et al., 2016; Koenig, 2020). The expectation is that individuals will fall back on their default emotion regulation strategies when invited to lay in a resting position and that this is what taps into resting vmHRV. A 5 min. resting measure of vmHRV has shown high to excellent test-retest correlations (Sinnreich et al., 1998; Tarkiainen et al., 2005; Li et al., 2009; Bertsch et al., 2012), supporting that resting levels reflect trait-like aspects of the typical interplay of the autonomic nervous system (Bertsch et al., 2012).

To control for circadian effects, collection of the ECG data occurred at approximately the same time in the afternoon for all participants (Bonnemeier et al., 2003). To further reduce other factors that might affect the activity of the heart, participants were asked to refrain from intake of nicotine, caffeine, and alcohol 3 h prior to the measurements. The ECG was recorded using disposable electrodes connected to a made-to-order ECG device (Research and Transfer Center at the University of Applied Sciences Leipzig, Germany) at a 1,000 Hz sampling rate. ECG data were acquired under two conditions: during rest and during playback of an audio file with a mindfulness instruction. Each condition lasted approximately 5 min. The ECG data were preprocessed, using the following steps: (1) Slow-drift-components were removed, in line with the procedure proposed by Sörnmo and Laguna (2005), that is, low-pass filtered (with a cut-off frequency of 0.5 Hz), polynomial spline fitting with in the isoelectric line, and estimation-subtraction of frequency components representing line noise (i.e., 50 Hz). The calculation of vmHRV was performed using Kubios (Tarvainen et al., 2014). The R-peaks were detected using the lead V3, with the automated algorithm provided by Kubios. Seven erroneous and two unidentified identified R-peaks were corrected after a subsequent visual inspection of all the data. Data from one participant was excluded due to poor data quality. The Square root of the mean squared differences of successive RR interval values (RMSSD) was calculated as indicative of vmHRV. Respiration was indicated by the peak in high frequency band (HF) of the FFT-spectrum. The HF was defined as 0.14–0.40 Hz. The RMSSD scores were log transformed to approximate a normal distribution.

### Statistical Analysis

All statistical analyses were performed in SPSS, version 24. Preliminary statistical analyses were performed with independent *t*-test and bivariate correlational analyses including sample characteristics and vmHRV, depressive and ruminative tendencies symptom scores, and self-compassion scores. Multiple linear regression analyses were conducted to test the main hypotheses in that self-compassion scores were included as predictors and ruminative tendencies and vmHRV as outcome variables, respectively. Further, ruminative tendency scores were included as a predictor and vmHRV as an outcome variable. In follow-up linear regression analyses, the SCS subscales were included as predictors of ruminative tendencies and vmHRV. We wanted to explore the specific contribution of the positive and negative SCS subscales, respectively. In all the multiple linear regression analyses, we controlled for the effects of age, gender, BMI, and depressive symptom severity scores (BDI), and additionally for mean heart rate and HF peak when vmHRV was included as the outcome variable. In additional analyses, we repeated the multiple linear regression analyses to test for the effects of antidepressant medication and > 10 prior MDD episodes. Due to multiple hypotheses testing, we Bonferroni-corrected the alpha level according to the two hypotheses tested (0.05/2), giving a significance level of 0.025 in the current study. To ensure that our results were not due to method-specific variance, we repeated the multiple linear regression analyses with alternative measures for self-compassion (FSCRS scores), and ruminative tendencies (RRS Brooding scores; see Supplementary Material).

Since only a patient group with recurrent MDD was included in the current study, we expected skewness and variability in scores. We chose to be careful in transforming or replacing outlier scores due to a high variability in scores probably reflecting “true” variance among the participants. Outliers were defined as ± 3 standard deviations from the sample mean. Of the main variables of vmHRV (log transformed), SCS total scores, and RRQ total scores, we only found two scores more than three standard deviations under the mean total score of the RRQ (see Supplementary Figures 1, 2 for scatterplots of the relations between SCS total scores and RRQ total scores, and SCS total scores and vmHRV, respectively). We did not adjust these two outlier scores, instead we included in the statistical analyses in Supplementary Material an alternative measure of ruminative tendencies, RRS Brooding scores. The RRS Brooding scores did not contain any outliers. In the multiple linear regression analyses the Variance Inflation Factor (VIF) were below 2, except for when including the SCS subscale scores as predictors in follow-up analyses. The VIF was then between 2.3 and 2.9 suggesting that the results for each of the SCS subscale scores reflected shared variance with the other SCS subscale scores.

## Results

### Descriptive Sample Characteristics and Preliminary Analyses

The mean age in the sample of adults with recurrent MDD was 40.24 years old (*SD* = 12.83) and 73% were females. The mean BMI was 25.81 (*SD* = 4.82). There were no significant gender differences for age, BMI, vmHRV, depression severity (BDI total score), SCS (total or subscores), or RRQ (total score). The only significant gender difference was obtained for mean heart rate where males had higher values than females [*M*_*M*_ = 997.39, *M*_*F*_ = 910.02 *t*(61) = −2.14, *p* = 0.04]. Higher age was correlated with lower vmHRV, a lower HFpeak, higher HDRS scores, and lower scores on the Self-Kindness subscale of the SCS (see Table 1). On the other hand, age did not correlate with mean heart rate, self-reported depressive severity (BDI total score), RRQ (total score), or SCS (total or subscales scores except the Self-Kindness score, see above). Level of BMI did not correlate with age, vmHRV or any of the variables included in the study.

**TABLE 1:** Bivariate correlations between self-compassion, rumination, vmHRV, and covariates.

		2	3	4	5	6	7	8	9	10	11	12	13	14	15
1	Resting vmHRV (RMSSD)	0.13	0.17	0.09	0.09	–0.08	–0.03	–0.15	–0.08	−0.25*	−0.25*	–0.14	0.53**	−0.49**	–20
2	SCS (total score)		0.79**	0.75**	0.78**	−0.79**	−0.74**	−0.70**	−0.48**	−0.43**	–0.17	–0.06	0.12	–0.12	–0.16
3	SCS self-kindness			0.70**	0.74**	−0.56**	−0.35**	−0.34**	−0.37**	−0.26*	–0.04	0.15	–0.08	−0.31*	–0.19
4	SCS common humanity				0.74**	−0.42**	−0.35**	−0.27*	–0.20	−0.37**	–0.01	0.02	0.04	–0.13	–0.19
5	SCS mindfulness					−0.44**	−0.34**	−0.39**	–0.21	–0.18	0.01	0.10	0.01	–0.23	–0.16
6	SCS self-judgment						0.59**	0.55**	0.41**	0.30*	0.14	0.16	–0.15	–0.06	0.22
7	SCS isolation							0.61**	0.44**	0.49**	0.24	0.17	–0.14	–0.07	0.01
8	SCS over-identification								0.53**	0.31*	0.33**	0.17	–0.25	0.08	–0.04
9	RRQ rumination									0.19	0.06	0.01	–0.07	–0.04	0.06
10	BDI (Total score)										0.41**	0.04	–0.18	0.05	0.20
11	HDRS (Total score)											0.02	–0.17	0.26*	0.10
12	HF peak												–0.19	−0.26*	0.01
13	Mean heart rate													0.09	–0.16
14	Age														0.05
15	BMI														

N = 63. SCS, Self-Compassion Scale; RRQ-Rum, Rumination subscale of the Rumination-Reflection Questionnaire; resting vmHRV (RMSSD), vagally mediated heart rate variability, square root of the mean squared differences of successive RR interval values, during rest; BDI, Beck Depression Inventory; HDRS, Hamilton Depression Rating Scale; HF peak, Peak frequency of the high-frequency band (0.14–0.40 Hz). *p < 0.05; **p < 0.01.

In the sample of recurrent MDD, 18 adults used antidepressant medication. Further medication was used predominantly by the older study participants: four used blood thinning medication, six used blood pressure medication, and five females used estrogen. Administrating the M.I.N.I. Neuropsychiatric Interview showed that in the current sample none of the participants reported suicidal risk or ongoing mania, hypomania, bipolar disorders, obsession/compulsion disorder, PTSD, psychotic or eating disorders, or disorders of alcohol or substance dependences. Comorbid anxiety disorders were present as expected (see Hasin et al. (2018)); ongoing and lifetime panic disorder (*n* = 16), agoraphobia (*n* = 9), social anxiety disorder (*n* = 4), and generalized anxiety disorder (*n* = 12). The majority of the sample had experienced less than 10 MDD episodes (*n* = 38), whereas 25 had experienced 10 or more episodes.

Bivariate correlation analyses showed that lower vmHRV associated with higher depressive symptom severity scores on BDI and HDRS (see Table 1). Further, vmHRV correlated positively with mean heart rate. Level of vmHRV was not associated with level of SCS. Higher SCS total scores correlated with lower self-reported depressive symptom severity on BDI and lower ruminative tendencies scores on RRQ, and not with clinician-rated depression severity on HDRS. The depressive symptom severity scores from BDI and HDRS correlated with each other, whereas none of these scores correlated with ruminative tendencies as self-reported on the RRQ. The SCS total and subscale scores of Self-kindness, Self-judgment, Isolation, and Overidentification correlated with the RRQ total scores. The SCS subscale scores of Common humanity and Mindfulness did not correlate with the RRQ total scores. In Supplementary Material, bivariate correlations between the SCS scores and the compassion questionnaire of FSCRS subscale scores of Reassured self, Inadequate self, and Hated self all correlated with the exception that the Hated self from FSCRS did not correlate with the Mindfulness or Self-kindness scores from SCS (see Supplementary Table 3). Further, higher RRQ total scores correlated with higher self-reported ruminative tendencies on the RRS Brooding subscale (*r* = 0.30, *p* = 0.02).

### Associations Between Self-Compassion, Rumination, and Vagally Mediated Heart Rate Variability

To test the expectation that higher self-compassion would be associated with lower ruminative tendencies, we conducted multiple linear regression analyses including the RRQ total scores as the outcome variable and the SCS scores as predictors. We found that higher SCS total scores were associated with lower RRQ total scores (see Table 2). In follow-up regression analyses, we calculated two further models where one included the three positive SCS subscale scores and the other model included the three negative SCS subscales scores, respectively. Among the negative subscales only the Overidentification subscale scores were associated with higher RRQ total scores, whereas the regression model including the positive subscales as predictors was not significant. In all the linear regression analyses, we controlled for the effects of age, gender, BMI, and BDI total scores. None of these covariates were associated with level of RRQ total scores.

**TABLE 2:** Multiple regression analyses of the relation between self-compassion, rumination and vmHRV.

Outcome variable	Predictor	B (95% CI)	*R* ^2^	*df*	β
Rumination-RRQ	Age	−0.00(−0.02to0.01)			–0.10
	Gender	−0.06(−0.37to0.26)			–0.04
	BMI	−0.00(−0.03to0.03)			–0.02
	BDI-total score	−0.00(−0.02to0.02)			–0.02
	SCS-total score	−0.48(−0.73to−0.23)	0.24*	5/62	−0.50*
Rumination-RRQ	Age	−0.01(−0.02to0.00)			–0.18
	Gender	−0.05(−0.39to0.29)			–0.04
	BMI	−0.00(−0.04to0.03)			–0.03
	BDI-total score	0.01(−0.01−*to*0.03)			0.14
	SCS self-kindness	−43(−0.74to−0.12)			−0.54*
	SCS common humanity	0.12(−0.18to0.42)			0.17
	SCS mindfulness	0.04(−0.31to0.39	0.19	7/62	0.05
Rumination-RRQ	Age	−0.00(−0.01to0.01)			–0.06
	Gender	−0.02(−0.32to0.29)			–0.01
	BMI	0.01(−0.02to0.04)			0.06
	BDI-total score	−0.00(−0.02to0.02)			–0.04
	SCS self-judgment	0.06(−0.14to0.27)			0.09
	SCS isolation	0.09(−0.12to0.30)			0.15
	SCS overidentification	0.32(0.08−0.56)	0.32*	7/62	0.41*
Resting vmHRV (RMSSD)	Age	−0.01(−0.02to−0.01)			−0.58*
	Gender	−0.08(−0.19to0.04)			–0.11
	BMI	−0.01(−0.02to0.01)			–0.09
	Mean heart rate	0.00(0.00−0.00)			0.54*
	HF peak	−1.10(−2.09to0.11)			–0.19
	BDI-total score	−0.01(−0.01to0.00)			–0.15
	SCS-total score	−0.05(−0.14to0.04)	0.64*	7/62	–0.10
Resting vmHRV (RMSSD)	Age	−0.01(−0.02to−0.01)			−0.57*
	Mean heart rate	0.00(0.00−0.00)			0.56*
	HF peak	−0.1.15(−2.15to−0.14)			–0.20
	SCS self-kindness	0.10(−0.02to0.21)			0.23
	SCS common humanity	−0.02(−0.12to0.08)			–0.05
	SCS mindfulness	−0.07(−0.19to0.05)	0.63	6/62	–0.16
Resting vmHRV (RMSSD)	Age	−0.01(−0.02to−0.01)			−0.60*
	Mean heart rate	0.00(0.00−0.00)			0.56*
	HF peak	−1.15(−2.18to−0.12)			–0.20
	SCS self-judgment	−0.02(−0.10to0.06)			–0.06
	SCS isolation	0.01(−0.07to0.08)			0.02
	SCS overidentification	0.04(−0.05to0.13)	0.61	6/62	0.10

N = 63. SCS, Self-compassion scale, RRQ-Rum, Rumination subscale of the Rumination-Reflection Questionnaire; resting vmHRV, vagally mediated heart-rate variability, square root of the mean squared differences of successive RR interval values, during rest; BDI, Beck depression inventory; HF peak, Peak frequency of high-frequency band (0.14–0.40 Hz). 95% CI, 95% Confidence interval. * < 0.025 (Bonferroni corrected alpha level).

To test the expectation that higher self-compassion would be associated with higher vmHRV, we included vmHRV as the outcome variable and self-compassion as predictors in multiple linear regression analyses. The SCS total score was not associated with vmHRV. In follow-up regression analyses with the SCS subscale scores, neither the positive nor the negative SCS subscale scores were associated with vmHRV. We controlled for the effects of age, gender, BMI, mean heart rate, HFpeak, and BDI total scores in these linear regression analyses. Higher age and higher mean heart rate were associated with lower vmHRV, whereas BMI, gender, BDI total scores, or HF peak were not associated with level of vmHRV.

In all the multiple linear regression analyses, we additionally tested for the effects of antidepressant medication and the dichotomous variable of either having had more than 10 prior MDD episodes or not. Including these variables as covariates did not change the significant results reported above. However, use of antidepressant medication covaried with level of vmHRV in the regression models including the SCS total scores [β = −0.27, *t*(54) = −2.6, *p* = 0.014] and the negative SCS subscale scores [β = −0.22, *t*(52) = −2.6, *p* = 0.011], respectively. Further, having more or less than 10 prior episodes did not covary with neither the RRQ total scores nor vmHRV and use of antidepressant did not covary with the RRQ total scores.

Additionally, we also tested the association between self-compassion and ruminative tendencies and self-compassion and vmHRV using the FSCRS scores of Self-reassurance, Inadequate self, and Hated self. Higher FSCRS scores of Inadequate Self associated with higher RRQ total scores. The Self-reassurance and the Hated Self score did not associate with RRQ total scores and further, none of the FSCRS scores associated with level of vmHRV (see Supplementary Table 4). The linear regression analyses were also repeated with the RRS Brooding scores instead of the RRQ total scores, and again, higher ruminative tendencies associated with lower Self-Compassion total scores and not with vmHRV (Supplementary Table 5).

## Discussion

The aim of the current study was to investigate how self-compassion associates with rumination and vmHRV in adults with recurrent MDD. We expected that in adults with recurrent MDD, self-compassion would be (1) negatively associated with ruminative tendencies, (2) positively associated with vmHRV. The first hypothesis was confirmed, and we found that self-compassion was negatively associated with ruminative tendencies in recurrent MDD. The second hypothesis was not confirmed; contrary to expectation we did not find that self-compassion was associated with level of vmHRV.

Our finding that higher levels of self-compassion was associated with lower levels of rumination in adults with recurrent MDD corresponds with expectations (Neff, 2003a; Gilbert, 2009) and previous research in healthy, young adults (Neff, 2003a; Neff et al., 2007) and in adults with mental symptom disorders (Krieger et al., 2013; Karl et al., 2018; Bakker et al., 2019). Self-compassion and rumination can be regarded as mutually exclusive ways of meeting difficulties. Self-compassion involves kindness, mindful flexibility, and a broad perspective of human suffering. In contrast, rumination involves criticism, being stuck in negative repetitive thinking patterns and personal identification with difficulties in which one feels isolated and alone. Thus, being self-compassionate in times of stress or struggle is suggested to be linked to a sense of calm and security (Gilbert and Irons, 2004; Gilbert, 2009), whereas ruminating is linked to exaggeration and prolongation of negative mood. Tending to meet oneself with compassion and soothing in difficult times may thus counteract ruminative tendencies and dwelling on negative emotions.

Despite the association between self-compassion and ruminative tendencies in our sample, we did not find any association between self-compassion and level of vmHRV. Given previous research findings of a positive association between self-compassion and level of vmHRV the null finding in the present study was contrary to our à priory expectations. However, we are to our knowledge the first study to investigate the effects of self-compassion on vmHRV in sample of recurrent MDD. This is an important factor since recurrent MDD is reflecting a chronic course of depression and thereby typically show a higher vulnerability for lower vmHRV. Moreover, in previous research, the positive effect of self-compassion on vmHRV have predominantly been studied in younger adults (e.g., Arch et al., 2014; Matos et al., 2017; Petrocchi et al., 2017; Luo et al., 2018; Kirschner et al., 2019), as opposed to the relatively older sample in our study (mean age = 40.24 years old). Our findings of an association between self-compassion and ruminative tendencies, and no association between self-compassion and vmHRV, were confirmed in Supplementary Material when including alternative measures of self-compassion (FSCRS) and ruminative tendencies (RRS Brooding).

It is not only the autonomic nervous system that is considered to play a crucial role in stress responsivity in the body. Also, the HPA axis is highly involved in stress responses and is known to be prominent in the etiology of depression (Pariante and Lightman, 2008). In MDD, the HPA axis is overactive and stimulates the release of higher levels of the main stress hormone of cortisol when compared with healthy controls (Otte et al., 2016). Interestingly, in a recent study it has been shown that stimulating the HPA axis with metyrapone to increase levels of cortisol led to a reduced vagal tone in lower vmHRV (Agorastos et al., 2019). The opposite, stimulating suppression of cortisol production via the HPA axis, did not affect level of vmHRV. Based on the interaction between the two stress systems in the body, which probably affects each other negatively in a chronic course of MDD, the prefrontal lobes of the brain and the vagal nerve may not be involved in the regulation of the beat-to-beat intervals of the heart as we would find in healthy/non-depressed samples. The assumption behind using vmHRV as a marker of psychopathology is that it reflects the brain’s ability to regulate behavior and emotions via the prefrontal cortices (see Thayer and Lane (2009)). The HPA axis is, however, mainly regulated by the hippocampus and the pituary gland, which are medial, subcortical brain structures (Jacobson and Sapolsky, 1991). In a recent study (Agorastos et al., 2020), adults with MDD were compared on whether they had one or several prior depressive episodes on HPA axis and vmHRV. The results showed that with higher HPA axis stimulation for producing cortisol a lower vagal activity (vmHRV) appeared in the group with recurrent MDD and an opposite effect showed in the first episode MDD group with increasing vmHRV following higher levels of cortisol. The latter study may support a different role of vmHRV in recurrent MDD compared to in a life-time history/one-episode MDD. If abnormal HPA axis functioning is leading to lower vmHRV specifically in recurrent depression, this can explain why self-compassion did not associate with vmHRV in the current study. Thus, the results might have been different if we had included participants with less than three depressive episodes, as the depressive pattern may then not have become as established.

Another complicating factor when studying vmHRV in recurrent MDD, is that a “chronic” course of MDD is likely to adversely affect both mental and somatic health to a greater extent than one-episode MDD and healthy samples. Such health issues can influence the autonomic nervous system and act as “third” variables in the association between self-compassion and vmHRV. For instance, in our clinical sample, several important potential confounding variables can have affected the autonomic nervous system, such as cardiovascular diseases, high blood pressure, level of depressive symptoms, a wide age distribution, use of medication such as antidepressants, BMI, physical activity, and adverse life experiences. Age is known be an important predictor of vmHRV (Antelmi et al., 2004), and in our sample age was a strong predictor for the level of vmHRV and seemed to “conquer” all other effects. With increasing age, bodily processes change, and the prevalence of physical conditions generally increase (Kessler et al., 2010). One may also speculate that in a chronically depressed sample age could be an epiphenomenon of length of depression. We also controlled for the effects of gender, level of depression, BMI, and medication, but found none of these variables to be consistently associated with vmHRV. Nevertheless, these different potential confounding factors probably leads to heterogeneity in autonomic nervous system functioning in our sample of recurrent MDD. As such, a higher number of patients with recurrent MDD may be required to have sufficient statistical power to show significant effects of psychological factors on vmHRV.

Several other limitations of our study also need to be considered when interpreting the study findings: (1) The study has a cross-sectional design which does not allow us to draw conclusions regarding causality. Future research using longitudinal designs are thus necessary to gain more knowledge about the causalities of the relationship between self-compassion, rumination, and vmHRV. Still, cross-sectional findings are important first steps and may inform more resource demanding longitudinal designs. (2) Our study used a convenience sample, which could cause sampling bias. (3) Our study did not include a control group. Including a control group could have provided comparable information about relations between self-compassion, rumination, and vmHRV in a healthy sample. This is especially important considering the finding that age was a strong predictor in our regression models. In our study sample with recurrent MDD, age might have been confounded with the number of depressive episodes (relapses) and it could be important in future research to untangle the contribution of these two factors. (4) Although the sample was recruited to be in remission from MDD, at the point of testing, some adults still self-reported high on BDI. However, this has probably not affected our results since we controlled for level of self-reported symptoms of depression (BDI) in the regression analyses. (5) To measure trait levels of vmHRV we used a resting condition, which may tap a state condition in addition to trait levels (Bertsch et al., 2012). (6) The measurement of level of self-compassion, ruminative tendencies, and depression were based on self-reports, which are prone to several biases. Rating oneself on these factors can be a difficult cognitive task and is highly based on retrospective memory and self-insight into relevant thoughts emotions and behavior (Schwarz and Oyserman, 2001). Thus, participants’ self-ratings may represent their own perceptions of levels of self-compassion, rumination, and depression rather than “objective” or true levels. Self-reports may also be influenced by social desirability. On the other hand, a strength of the current study is that the results did not rely solely on self-report measures but were complemented by psychophysiological measures (which are affected by such biases to a very little degree, if at all). (7) Optimally, however, the current study could also have included alternative biological measures other than vmHRV, such as cortisol level or salivary alpha-amylase. Previous studies using healthy samples have found that higher levels of self-compassion significantly associates with lower cortisol levels (Herriot et al., 2018), lower blood inflammation (interleukin-6; Breines et al., 2014), and lower salivary alpha amylase concentration (another marker of sympathetic nervous system activation; Breines et al., 2015), and including such measures could add valuable knowledge about self-compassion and biological processes underlying stress responding in recurrent MDD. (8) To lower the probability of type I error, we controlled for multiple testing using Bonferroni correction. This is a conservative procedure that can increase the likelihood of type II error. (9) Lastly, our sample had few comorbid disorders compared to what is common in clinical-recruited samples of recurrent MDD (Steffen et al., 2020). This is a strength in studying the effect of self-compassion on vmHRV in relation to depression. However, at the same time, this can limit the generalizability of the findings in the current study to the typical samples of recurrent depression with comorbid disorders such as post-traumatic stress disorder (PTSD; Rytwinski et al., 2013). PTSD could for instance be a factor in recurrent depression influencing the relationship between self-compassion and vmHRV.

In summary, our study findings show that in a recurrent MDD sample, higher self-compassion significantly associates with lower ruminative tendencies, but does not associate with level of vmHRV. Rumination is recognized as a key vulnerability factor for depressive relapse, and thus an implication of the current study is that strengthening self-compassion is beneficial for individuals suffering from recurrent MDD. Although self-compassion at baseline in the current study was not associated with level of vmHRV, it is possible that systematic training in increasing self-compassion in recurrent MDD would lead to lower vmHRV. This is an interesting subject for further research, and could be examined for instance through subjecting individuals with recurrent MDD to programs designed to systematically increase self-compassion (e.g., Mindful Self-Compassion; Neff and Germer, 2013). Another implication of the current study is that age may be an important factor to take into consideration when performing clinical research on recurrent MDD. Based on the current results it seems beneficial to aim for relatively homogeneous age groups when conducting research on these variables, as participants will likely resemble each other more and show less variability on outcome measures. This may be particularly important for studies examining psychophysiological variables such as vmHRV.

## Data Availability Statement

The raw data supporting the conclusions of this article will be made available by the authors, without undue reservation.

## Ethics Statement

The studies involving human participants were reviewed and approved by the Regional Committee for Medical Research Ethics of South East Norway (Reference: 2016/388). The patients/participants provided their written informed consent to participate in this study.

## Author Contributions

LS and ES: project leaders. JS, ES, BO, JV, EV, SJ, and LS: conception and design and collection of the data. JS, BO, and LS: analysis and interpretation. LS and JS: writing of the manuscript. AK, ES, BO, JV, EV, P-EB, and SJ: critical review of the manuscript. JS, ES, EV, SJ, JV, AK, BO, P-EB, and LS: final approval for publication. All authors contributed to the article and approved the submitted version.

## Conflict of Interest

The authors declare that the research was conducted in the absence of any commercial or financial relationships that could be construed as a potential conflict of interest.

## Publisher’s Note

All claims expressed in this article are solely those of the authors and do not necessarily represent those of their affiliated organizations, or those of the publisher, the editors and the reviewers. Any product that may be evaluated in this article, or claim that may be made by its manufacturer, is not guaranteed or endorsed by the publisher.

## Footnotes

1
https://mindfulness.w.uib.no/
