# Supplementary material for: Self-Compassion and Its Association With Ruminative Tendencies and Vagally Mediated Heart Rate Variability in Recurrent Major Depression
Source: Front Psychol. 2022 Mar 7;13:798914. doi: 10.3389/fpsyg.2022.798914 (PMC8940525; doi:10.3389/fpsyg.2022.798914)
Supplement: Supplementary file 1 [file Data_Sheet_1.PDF]

Supplemental figures

Supplemental Figure 1: Scatterplot of the association between SCS and RRQ-rum

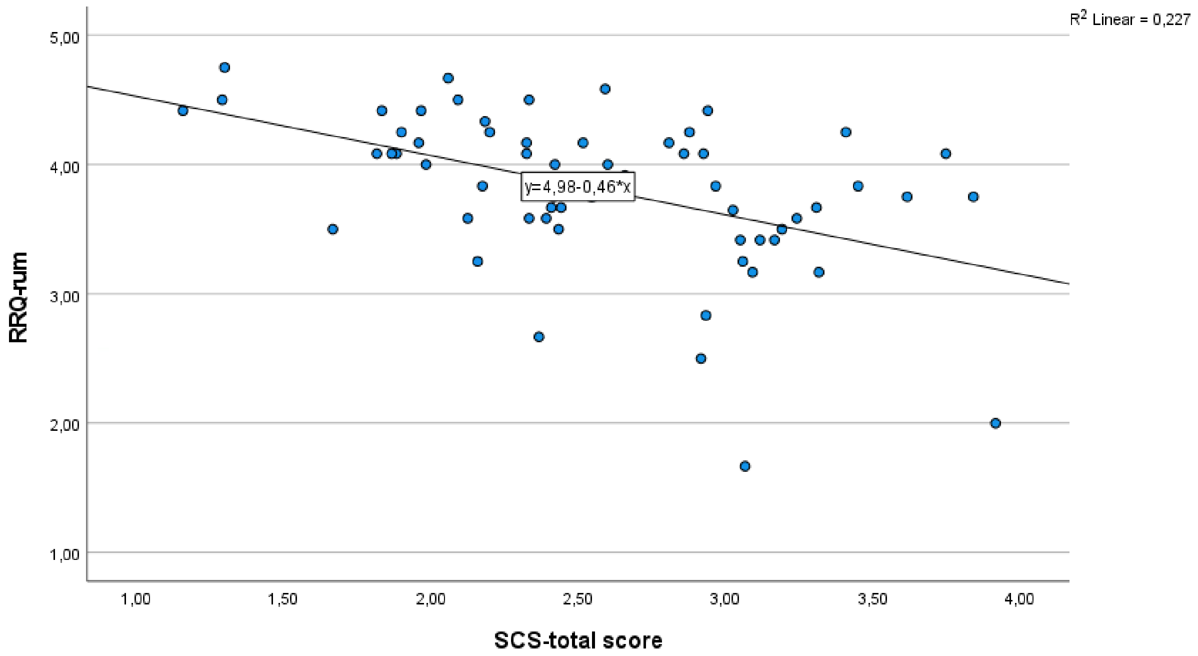

Note: *N* = 63.SCS total score = total score of the Self-compassion scale; RRQ-Rum= total score of the Rumination subscale of the Rumination-Reflection Questionnaire

Supplemental Figure 2: Scatterplot of the association between SCS and vmHRV

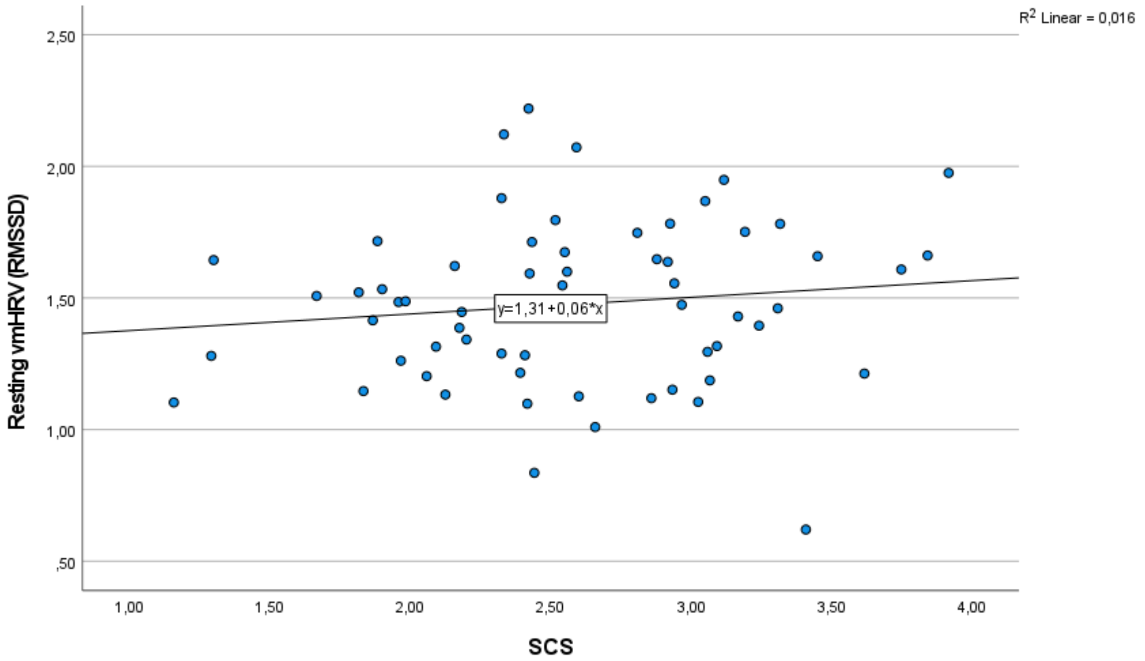

Note: *N* = 63. SCS total score = total score of the Self-compassion scale
