# Supplementary material for: Self-Compassion and Its Association With Ruminative Tendencies and Vagally Mediated Heart Rate Variability in Recurrent Major Depression
Source: Front Psychol. 2022 Mar 7;13:798914. doi: 10.3389/fpsyg.2022.798914 (PMC8940525; doi:10.3389/fpsyg.2022.798914)
Supplement: Supplementary file 2 [file Table_1.pdf]

Supplemental tables

Supplemental table 1: Inter-item correlation table for SCS subscales

SCS Self-kindness: 5 items

	Item 5	Item 12	Item 19	Item 23	Item 26
Item 5	1.00				
Item 12	.58	1.00			
Iitem 19	.63	.67	1.00		
Item 23	.40	.50	.57	1.00	
Item 26	.45	.39	.57	.70	1.00

SCS Self-judgment: 5 items

	Item 1	Item 8	Item 11	Item 16	Item 21
Item 1	1.00				
Item 8	.27	1.00			
Item 11	.49	.30	1.00		
Item 16	.66	.44	.47	1.00	
Item 21	.52	.53	.57	.57	1.00

SCS Common humanity: 4 items

	Item 3	Item 7	Item 10	Item 15
Item 3	1.00			
Item 7	.57	1.00		
Item 10	.44	.74	1.00	
Item 15	.51	.63	.60	1.00

SCS Isolation: 4 items

	Item 4	Item 13	Item 18	Item 25
Item 4	1.00			
Item 13	.50	1.00		
Item 18	.50	.59	1.00	
Item 25	.61	.54	.47	1.00

SCS Mindfulness: 4 items

	Item 9	Item 14	Item 17	Item 22
Item 9	1.00			
Item 14	.66	1.00		
Item 17	.39	.39	1.00	
Item 22	.21	.23	.09	1.00

SCS Over-identification: 4 items

	Item 2	Item 6	Item 20	Item 24
Item 2	1.00			
Item 6	.58	1.00		
Item 20	.34	.33	1.00	
Item 24	.56	.32	.34	1.00

Note: N = 63. SCS = Self-compassion scale

Supplemental table 2: Inter-item correlation table for RRQ-rum

	Item 1	Item 2	Item 3	Item 4	Item 5	Item 6	Item 7	Item 8	Item 9	Item 10	Item 11	Item 12
Item 1	1.00											
Item 2	.48	1.00										
Item 3	.59	.44	1.00									
Item 4	.55	.58	.54	1.00								
Item 5	.47	.41	.57	.48	1.00							
Item 6	.12	.33	.29	.39	.32	1.00						
Item 7	.59	.59	.57	.72	.54	.41	1.00					
Item 8	.57	.58	.55	.74	.42	.24	.75	1.00				
Item 9	.61	.55	.46	.44	.51	.33	.46	.43	1.00			
Item 10	.44	.38	.35	.38	.26	.50	.43	.26	.44	1.00		
Item 11	.58	.57	.59	.61	.68	.39	.65	.63	.54	.33	1.00	
Item 12	.51	.51	.51	.53	.60	.49	.60	.55	.55	.22	.74	1.00

Note: *N*= 63. RRQ-Rum= Rumination subscale of the Rumination-Reflection Questionnaire

Supplemental Table 3: Bivariate correlations between SCS and FSCRS

		FSCRS scores		
		Reassured Self	Inadequate Self	Hated Self
1	SCS (total score)	.61**	-.56**	-.49**
2	SCS Self-kindness	.59**	-.38**	-.19
3	SCS Common humanity	.42**	-.29**	-.32*
4	SCS Mindfulness	.43**	-.29*	-.23
5	SCS Self-judgment	-.48**	.58**	.52**
6	SCS Isolation	-.43**	.51**	.53**
7	SCS Over-identification	-.41**	.45**	.36**
8	FSCRS Reassured self		-.53**	-.45**
9	FSCRS Inadequate self			.63**
10	FSCRS Hated self			

Note: *N* = 63. SCS = Self-compassion scale; FSCRS = Forms of Self-Critising / Attacking and Self-Reassuring Scale.

* p < .05; ** p < .01.

Supplemental Table 4: Multiple regression analyses of the relation between FSCRS, RRQ-rum and vmHRV

**Outcome variable**	**Predictor**	R^2	***df***	***β***
RRQ-rum	Age			-.12
	Gender			-.05
	BMI			-.02
	BDI (total score)			.14
	FSCRS Reassured self			-.19
	FSCRS Inadequate self			.41*
	FSCRS Hated self	.21	7/62	-.30
Resting vmHRV (RMSSD)	Age			-.56*
	Gender			-.10
	BMI			-.08
	Mean heart rate			.53*
	HF peak			-.18
	BDI (total score)			-.12
	FSCRS Reassured self			-.06
	FSCRS Inadequate self			-.06
	FSCRS Hated self	.64	9/62	.02

Note: *N* = 63. FSCRS = Forms of Self-Critising / Attacking and Self-Reassuring Scale, RRQ-rum = RRQ-Rum, Rumination Subscale of the Rumination-Reflection Questionnaire. *< 0.025 (Bonferroni corrected alpha level)

Supplemental Table 5: Multiple regression analyses of the relation between RRS-brooding og vmHRV

**Outcome variable**	**Predictor**	R^2	***df***	***β***
RRS brooding	Age			-.29*
	Gender			-.07
	BMI			-.06
	BDI (total score)			.20
	SCS total	.30	5/62	-.38*
Resting vmHRV (RMSSD)	Age			-.55*
	Gender			-.10
	BMI			-.08
	Mean heart rate			.55*
	HF peak			-.20
	BDI (total score)			-.13
	RRS brooding	.64	7/62	.07

Note. $N = 63$. RRS Brooding = Brooding subscales of the Ruminative Response Scale. *< 0.025 (Bonferroni corrected alpha level)
